# Analysis of Physicochemical Properties, Lipid Composition, and Oxidative Stability of Cashew Nut Kernel Oil

**DOI:** 10.3390/foods12040693

**Published:** 2023-02-06

**Authors:** Yijun Liu, Leshi Li, Qiuyu Xia, Lijing Lin

**Affiliations:** 1 Hainan Key Laboratory of Storage & Processing of Fruits and Vegetables, Agricultural Products Processing Research Institute, Chinese Academy of Tropical Agricultural Sciences, Zhanjiang 524001, China; 2 Key Laboratory of Tropical Crop Products Processing of the Ministry of Agriculture and Rural Affairs, Agricultural Products Processing Research Institute, Chinese Academy of Tropical Agricultural Sciences, Zhanjiang 524001, China; 3 College of Food Science and Technology, Guangdong Ocean University, Zhanjiang 524088, China

**Keywords:** cashew nuts, oil, lipids, oxidative stability, physicochemical properties

## Abstract

Cashew nut kernel oil (CNKO) is an important oil source from tropical crops. The lipid species, composition, and relative content of CNKO were revealed using ultra high performance liquid chromatography time-of-flight tandem mass spectrometry (UPLC-TOF-MS/MS), and the physicochemical properties, functional group structure, and oxidation stability of CNKO at different pressing temperatures were characterized using a near infrared analyzer and other methods. The results showed that CNKO mainly consisted of oleic acid (60.87 ± 0.06%), linoleic acid (17.33 ± 0.28%), stearic acid (10.93 ± 0.31%), and palmitic acid (9.85 ± 0.04%), and a highly unsaturated fatty acid (78.46 ± 0.35%). In addition, 141 lipids, including 102 glycerides and 39 phospholipids, were identified in CNKO. The pressing temperature had a significant effect on the physicochemical properties of cashew kernels, such as acid value, iodine value, and peroxide value, but the change in value was small. The increase in pressing temperature did not lead to changes in the functional group structure of CNKO, but decreased the induction time of CNKO, resulting in a decrease in their oxidative stability. It provided basic data support to guide subsequent cashew kernel processing, quality evaluation, and functional studies.

## 1. Introduction

The cashew nut is a genus of plants of the genus Dicotyledonea, order Sapotaceae, family Lacertidae, and genus Cashew [1]. Currently, the countries with relatively large cashew cultivation areas in the world include India, Brazil, Vietnam, Côte d’Ivoire, Mozambique, and Tanzania, whose total output accounts for more than 60% globally. According to the statistics of the world food and agriculture organization (FAO), it can be seen that the total global production of cashew nuts (in shell) was up to 4,093,000 tons in 2018 and 3,960,700 tons in 2019. The total cashew nut production (in 2019) in Africa, represented by Côte d’Ivoire and Mozambique, was 2,334,400 tons, accounting for 60% of the world’s total production. The total production in Asia, represented by India, was 1,474,900 tons, accounting for 37%, and the total production in the Americas, represented by Brazil, was 151,400 tons, accounting for 3%.

Cashew nut kernels (CNKs) are the kernels of shelled cashew nuts after dehulling, which are mainly composed of 47.0% fat, 21.1% protein, 4.6–11.2% starch, 2.4–8.7% sugar, and other components, as well as a variety of amino acids, vitamins, and trace elements, such as phosphorus, iron, and calcium [1]. CNK processing mainly produces primary products, and deep processing products are less common, such as charbroiled cashew nuts, seaweed cashew nuts, salt-baked cashew nuts, cashew oil microcapsules [2], defatted cashew nut flour [3], and probiotic drinks [4]. In addition, Sravani et al. [5] added crushed CNKs to the feed of lambs to improve weight gain, feed efficiency, and economic meat production in male lambs. Morgane et al. [6] fed C. gariepinus with CNKO instead of fish oil and palm oil to achieve an increased net profit value (NPV) (23.15$) from a low investment. The results of Emelike et al. [7] showed that CNKO was effective in reducing serum cholesterol and triglyceride levels in rats.

CNKO is a very important nutritional component of CNKs, accounting for approximately 47% [8]. CNKO contains 11 saturated fatty acids, accounting for 25.37% of the total content, with palmitic acid (12.20%), stearic acid (11.30%), arachidic acid (1.07%), and behenic acid (0.22%). It also contains seven unsaturated fatty acids, accounting for 71.98% of the total content, with oleic acid (51.47%), linoleic acid (19.66%), palmitoleic acid (0.36%), and eicosanoic acid (0.34%) [9].

The aqueous extraction method, squeezing extraction, supercritical carbon dioxide extraction, and solvent extraction method were common extraction methods for oils. Phuong et al. [10] used enzyme-assisted aqueous extraction for cashew nut oil recovery of 86.28% with lower peroxide and free fatty acid values than those obtained by Soxhlet extraction. Li et al. [11] determined the optimal processing conditions for CNKO extraction using the aqueous extraction method: the temperature was set to 80 °C, the material–liquid ratio was 1:3, the centrifugal force was 6000 r/min, and the oil yield was 34.86%.

CNKs are rich in protein and tightly combined with oil, and the aqueous extraction method easily produced the serious emulsification phenomenon, which affects the extraction of oil. The supercritical carbon dioxide extraction method involves a large equipment cost investment, while the pressing method is a simple and low-cost process. However, there are few reports on the lipid composition of CNKO and the effect of pressing temperature on the physicochemical properties, functional group composition, and oxidative stability of CNKO. Therefore, in this study, the lipid species, composition, and relative content were revealed in CNKO using high performance liquid chromatography time-of-flight tandem mass spectrometry, and the physicochemical properties, functional group structure, and oxidation stability of CNKO at different pressing temperatures were characterized using a near infrared analyzer and other methods. This study will provide basic data for the processing and product development of CNKO.

## 2. Materials and Methods

### 2.1. Preparation of Cashew Nut Kernel Oil

Cashew nut kernels (W240) were purchased from Chang-da-Chang Super Shopping Supermarket, Zhanjiang, China. The temperature of the press was adjusted, and then the cashew kernels were placed in the press (LTP200, Dongguan Minjian Electric Industrial Co., Zhanjiang, China) for extraction, and the crude oil was collected and centrifuged at 20 °C and 5000 rpm for 10 min, and the upper oil layer was collected and stored at 4 °C for use.

### 2.2. Physicochemical Analysis of Cashew Nut Kernel Oil

The determination of acid values was made with reference to GB5009.229-2016 [12], and the determination of iodine values was made with reference to GBT5532-2008 [13].

The peroxide value was determined by referring to the method of Akbar et al. [14] with appropriate modifications. A total of 0.02 mg of oil (m) was dissolved in 2 mL of chloroform/methanol (7:3), 0.05 mL of ammonium thiocyanate (0.3 g/mL) was added as sample solution 1, and the absorbance of sample solution 1 at 501 nm was E_0_. A total of 0.05 mL of ferrous chloride was added to sample solution 1, and the reaction was left for 5 min after vortex shaking to obtain sample solution 2, and the absorbance of sample solution 2 was at 501 nm. The absorbance of sample solution 2 at 501 nm was E_2_. Without adding oil, the absorbance at 501 nm was E_1_, and the peroxide value (POV) = (E_2_ − (E_0_ + E_1_))/(55.82 × m).

The specific extinction coefficient was determined by referring to the method of HST57-2017 and Yakindra et al. [15] with appropriate modifications. A total of 0.25 g of oil sample was put into a test tube, dissolved by adding 5 mL of isooctane, and then diluted to 25 mL by adding isooctane. The concentration of the sample solution was ω (g/100 mL), and the oil sample was placed in the automatic refractometer (XFZGY-3000, Xiamen Xiongfa Instrument Co., Xiamen, China) for measurement. Using isooctane as the reference, the absorbance of the sample solution at 232 nm was A_232_, and the corresponding specific extinction coefficient K_232_ = A_232_/ω. The absorbance of the sample solution at 270 nm was A_270_, and the corresponding specific extinction coefficient K_270_ = A_270_/ω.

### 2.3. Determination of Fatty Acid Composition of Cashew Nut Kernel Oil

The fatty acid composition in cashew nut kernel oil was determined by gas chromatography (GC-MS) (LC-30A, Shimadzu, Kyoto, Japan) coupled with the potassium hydroxide methylation method. The relevant parameters were referred to in Liu et al. [16].

### 2.4. Determination of Lipids of Cashew Nut Kernel Oil

The lipid composition in CNKO oil was performed by a Shimadzu UPLC LC-30A system (LC-30A liquid chromatograph, Shimadzu Corporation, Kyoto, Japan) equipped with a Phenomenex Kinete C18 column (100 × 2.1 mm, 2.6 µm), and the relevant parameters were referred to in Liu et al. [16].

### 2.5. Determination of Fourier Transform Infrared Spectroscopy of Cashew Nut Kernel Oil

Referring to the method of Yakindra et al. [15] with some modifications, oil droplets were placed on the plane sensitive surface of the crystal and then placed in an infrared spectrometer (Thermo Nicolet iN10, ThermoFisher Scientific, Greenville, SC, USA) for measurement. The infrared spectra were collected in the range of 500–4000 cm^−1^ with a resolution of 4 cm^−1^ and 64 scans, and the infrared spectral data were collected with the OMSNIC software (Thermo Nicolet iN10, ThermoFisher Scientific, Greenville, SC, USA) with an average of 3 parallel acquisitions per sample. The average spectrum was used as the sample spectrum, and the infrared spectral curve was plotted using Origin software (2021, OriginLab Corporation, Northampton, UK).

### 2.6. Determination of Oxidative Stability of Cashew Nut Kernel Oil

The oxidative stability of the oil was determined by referring to the method of Xia et al. [17] with some modifications. A 3.5 g oil sample was weighed into a test tube, which was placed in an oil oxidation stability tester (Rancimat743, Swiss Aptar China Ltd., Hong Kong, China) at 110 °C with an air flow rate of 20 L/h. The induction time was recorded from the inflection point of the conductivity curve and the results were recorded in hours.

### 2.7. Data Processing and Analysis

All samples were measured 3 times in parallel. LipidView software (v2.0, ABSciex, Concord, ON, Canada) was used to undertake qualitative analysis of shotgun-MS data. In the process of data analysis, the relevant parameters of the software were set as follows: the mass tolerance was 0.5, the minimum% intensity was 1, the minimum signal-to-noise ratio was 10, the average flow injection spectrum from the top was 30% TIC, and the total double bond was ≤12. OriginPro (2021, OriginLab Corporation, Northampton, UK), SIMCA (14.1, Sartorius Lab Instruments GmbH & Co. KG, Goettingen, Germany), and Photoshop (2022, Adobe Systems Incorporated, San Jose, CA, USA) were used for plotting, data processing, and statistical analysis.

## 3. Results and Analysis

### 3.1. Analysis of Fatty Acid and Lipid Composition of Cashew Nut Kernel Oil

The fatty acids of CNKO were palmitic acid (9.85 ± 0.04%), palmitoleic acid (0.26 ± 0.01%), stearic acid (10.93 ± 0.31%), oleic acid (60.87 ± 0.06%), linoleic acid (17.33 ± 0.28%), and arachidonic acid (0.76 ± 0.02%). The content of saturated fatty acids was approximately (21.54 ± 0.37%) and unsaturated fatty acids was approximately (78.46 ± 0.35%), including (61.13 ± 0.07%) of monounsaturated fatty acids.

**Figure 1:**
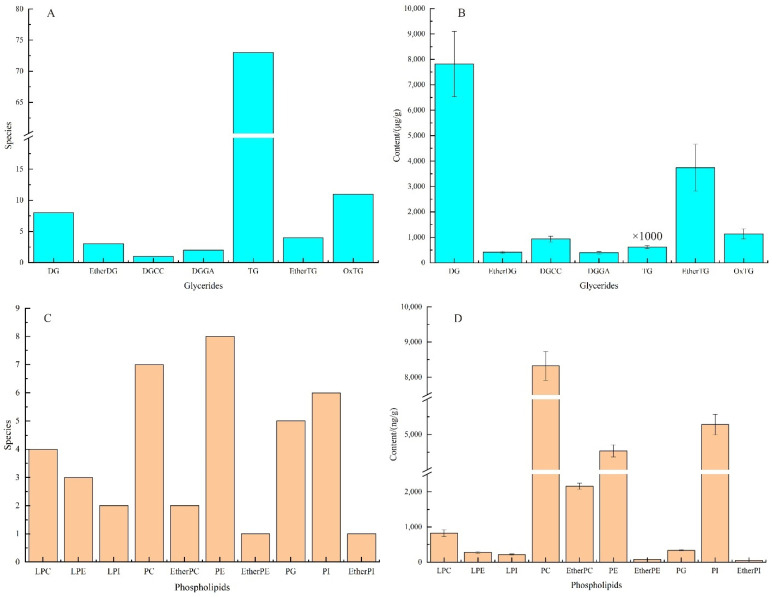
(**A**,**B**) represents the number and content of different phospholipids, (**C**,**D**) represents the number and content of different glycerides, respectively.

The lipid composition of CNKO was comprehensively profiled using UPLC-TOF-MS/MS, and information on the precise relative molecular masses of lipids, isotopic distribution, and secondary mass spectrometry cleavage fragments were obtained in composite scanning mode. The lipids of CNKO were identified as shown in Table 1 and Table 2 and Figure 1. As shown in Figure 1A,B, 141 lipids, composed of 102 glycerides, and 39 phospholipids, were identified in CNKO. The 102 glycerides mainly included 8 diacylglycerol (DG), 3 ether-linked diacylglycerol (EtherDG), 1 diacylglyceryl-3-O-carboxyhydroxy methylcholine (DGCC), 2 diacylglyceryl glucuronide (DGGA), 73 triglycerides (TG), 4 ether-linked triacylglycerol (EtherTG), and 11 oxidized triglycerides (OxTG). A total of 39 phospholipids mainly included 4 lysophophatidylcholine (LPC), 3 lysophosphatidylethanolamine (LPE), 2 lysophosphatidylinositol (LPI), 7 phosphatidylcholine (PC), 2 ether-linked phosphatidylcholine (EtherPC), 8 phosphatidylethanolamine (PE), 1 ether-linked phosphatidylethanolamine (EtherPE), 5 phosphatidylglycerol (PG), 6 phosphatidylinositol (PI), and 1 ether-linked phosphatidylinositol (EtherPI).

As shown in Table 1, the total number of carbon atoms in the fatty acid side chains of lipids in CNKO was 16–32, and the double bond number was 0–7. DG in glyceride has 32–38 carbon atoms and 0–4 double bonds and the side chains were mainly composed of C16, C18, and C20. EtherDG had 34–36 carbon atoms, with a double bond number of 2–4 and the side chains were mainly composed of C15, C17, and C19. DGCC had 36 carbon atoms, with a double bond number of 2. DGGA had 34–36 carbon atoms, with a double bond number of 1–2 and the side chains were mainly composed of C16 and C18. TG had 34–36 carbon atoms, with a double bond number of 0–7 and the side chains were mainly composed of C8, C10, C12, C14, C15, C16, C17, C18, C20, C21, C22, C23, C24, C25, and C26. EtherTG had 53–55 carbon atoms, with a double bond number of 2–5 and the side chains were mainly composed of C16, C17, C18, and C19. OxTG had 50–56 carbon atoms, with a double bond number of 2–6 and the side chains were mainly composed of C16, C18, and C20. As shown in Table 2, LPC and LPE in phospholipids had 16–18 carbon atoms, with a double bond number of 0–2. LPI had 16–18 carbon atoms, with a double bond number of 0–1. PC had 32–36 carbon atoms, with a double bond number of 0–4, and the side chains were mainly composed of C16 and C18. EtherPC had 34–37 carbon atoms, with a double bond number of 1, and the side chains were mainly composed of C16, C17, and C21. PE had 32–36 carbon atoms, with a double bond number of 0–4, and the side chains were mainly composed of C16 and C18. EtherPE had 40 carbon atoms, with a double bond number of 5, and the side chains were mainly composed of C18 and C22. PG had 32–36 carbon atoms, with a double bond number of 0–2, and the side chains were mainly composed of C16 and C18. PI had 34–36 carbon atoms, with a double bond number of 1–2, and the side chains were mainly composed of C16 and C18. EtherPI had 18 carbon atoms, with a double bond number of 0.

As shown in Figure 1C, it could be seen that the content of each glyceride in CNKO was ranked as TG > DG > EtherTG > OxTG > DGCC > EtherDG > DGGA, where TG had (617.97 ± 60.02) mg/g and DG had (7.82 ± 1.28) mg/g. As shown in Figure 1D, it could be seen that the content of each phosphate ester in CNKO was ranked as PC> PI > PE > EtherPC > LPC > PG > LPE > LPI > EtherPE > EtherPI, where PC had (8.32 ± 0.41) μg/g and PI had (5.28 ± 0.29) μg/g.

### 3.2. Physicochemical Properties of Cashew Nut Kernel Oil

The CNKO obtained at different pressing temperatures was slightly yellow in color, without obvious precipitation, with a slight aroma of cashew nut kernel, and its physicochemical properties are shown in Table 3. As can be seen from Table 3, the acid value of cashew nut oil was (0.41–0.53) mg/g < 4 mg/g as national standard and the peroxide value was (0.036–0.118) g/100 g < 0.25 g/100 g as national standard, all of which satisfied GB 2716–2018 “National Standard for Food Safety Vegetable Oil” [18]. The specific extinction coefficient at 232 nm was related to the primary and secondary stages of oil oxidation, while the specific extinction coefficient at 270 nm was related to the secondary stages of oil oxidation [19,20]. The K232 and K270 of CNKO were (1.0–1.2) and (0.05–0.12), respectively, indicating that the cashew nut kernel oil obtained using the pressing method contains only a very small amount of hydroperoxides and was not easily acidified.

The results of the significance analysis showed that there were different degrees of influence of pressing temperature on acid value, iodine value, peroxide value, refractive index, and specific extinction coefficient. The differences in acid value were not significant at temperatures greater than 100 °C. The differences in peroxide value were not significant at 140 °C and 160 °C, and significant at other temperatures (100 °C, 120 °C, 160 °C, 180 °C). The differences in iodine value were not significant at 120 and 140 °C, and significant at other temperatures. The differences in the refractive index were not significant at 160°C and 180 °C, and the differences in the refractive index were not significant at 100 °C, 120 °C, 140 °C, and 200 °C, and significant between the two groups. Although the results of the significance analysis showed that the relevant indexes of the oils obtained at different temperatures were affected, the value fluctuated less, indicating that the physicochemical properties of CNKO were relatively stable.

### 3.3. Near-Infrared Spectral Characteristics of Cashew Nut Kernel Oil

**Figure 2:**
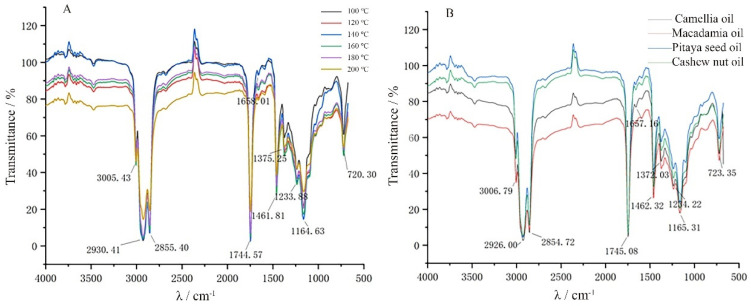
NIR spectral characteristics of cashew nut kernel oil. (**A**) represents the NIR spectral characteristics of CNKO at different pressing temperatures; (**B**) represents the NIR characteristics of CNKO compared with other oils.

The Fourier NIR spectra of CNKO are shown in Figure 2. Figure 2A represents the NIR spectra of cashew nut kernel oil at different pressing temperatures, and Figure 2B represents the NIR spectra of different types of oils.

As shown in Figure 2A, it can be seen that the peak at 3005.43 cm^−1^ corresponds to the -CH_3_ antisymmetric stretching vibration, at 2930.41 cm^−1^ corresponds to the -CH_2_ antisymmetric stretching vibration, at 2855.40 cm^−1^ corresponds to the -CH_2_ symmetric stretching vibration, and at 1744.57 cm^−1^ corresponds to the C=O stretching vibration. This could be used to identify ketones, aldehydes, acids, esters, and anhydrides. The possibility that CNKO had an anhydride structure was ruled out because the anhydride would have a double peak due to vibrational coupling. Although 1658.01 cm^−1^ corresponded to the C=C stretching vibration, 2–4 peaks due to benzene ring skeleton vibration were not found near 1600 cm^−1^ and 1500 cm^−1^, so the possibility of the presence of an aromatic ring structure in CNKO was excluded. The peak at 1461.81 cm^−1^ corresponded to the -CH_3_ asymmetric deformation vibration and at 1375.25 cm^−1^ corresponded to the -CH_3_ symmetric deformation vibration. Although it corresponded to the C-C stretching vibration at 1233.88 cm^−1^, the position of this absorption band changed with the structure of the compound molecule due to the vibrational coupling effect and weak intensity, so it was not meaningful in the structure identification. The peak at 1164.63 cm^−1^ corresponded to the C-O stretching vibration in alcohols, which could be used to distinguish between primary, secondary, and tertiary alcohols, and CNKO might be the C-O stretching vibration of tertiary alcohols. The peak at 720.30 cm^−1^ corresponded to a swinging vibration in the -CH_2_ plane and had more than four methylene-linked structures. As shown in Figure 2B, it can be seen that the NIR spectra of camellia seed oil, macadamia nut oil, pitaya seed oil, and cashew nut kernel oil had similar peak shapes, peak positions, and number of characteristic peaks, indicating that the functional groups of the oils had similar structures. However, the peak signal intensities of different oils and fats were different, probably due to differences in the number of functional groups, such as differences in the composition of the oils [21].

### 3.4. Analysis of Oxidative Stability of Cashew Nut Kernel Oil

Oxidative stability could be a good predictor of the oxidation reaction of oils. The auto-oxidation of oils was divided into the induction phase as well as the oxidation phase, and the length of time required from the induction phase to the oxidation phase could reflect the ability of oils to resist auto-oxidation, which was the oxidation stability of oils [22,23]. The induction time could indirectly reflect the size of the oxidative stability of oils. The longer the induction period was extended, the better the oxidative stability of oils, and vice versa, the worse their oxidative stability.

**Figure 3:**
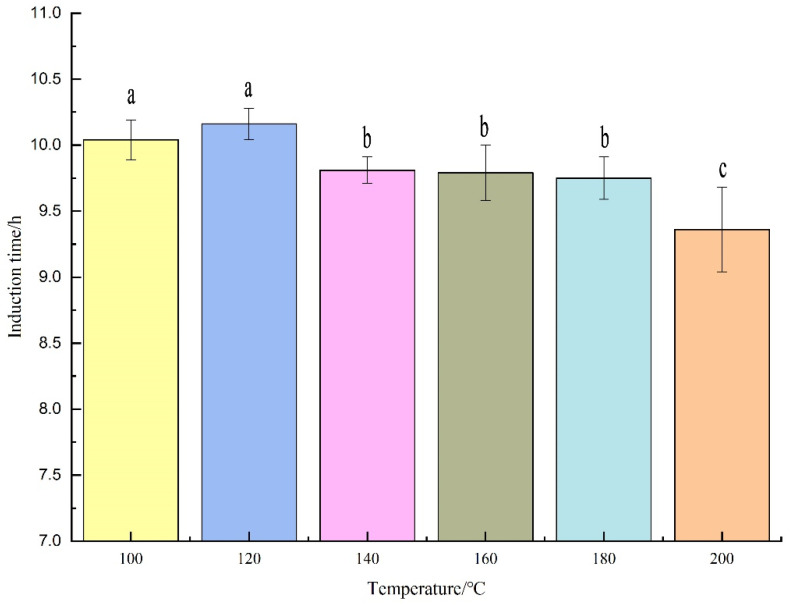
Induction time of cashew nut kernel oil obtained from different pressing temperatures. Note: Different letters a, b and c represented significant differences.

As shown in Figure 3, the oxidative stability indices of CNKO at different pressing temperatures were in the interval of 9.3–10.2 h with an error of no more than 1 h. Meanwhile, the oxidative stability indices began to show a decreasing trend when the pressing temperature was greater than 120 °C. The results of the significance analysis showed that the induction time of CNKO was not significant between 100 °C and 120 °C, and between 140 °C, 160 °C, and 180 °C. The difference between 200 °C and other temperatures was significant. It could be inferred that the oxidation rate of CNKO accelerated, and the induction time decreased as the extraction temperature increased, and the oxidative stability decreased.

## 4. Discussion

Cashew nut kernel oil accounted for approximately 47% of the cashew nut kernel content, which was higher than the oil content of avocado (8–29%) [24,25], olive (18–24%) [26], and camellia seed (14–27%) [27], etc., and lower than the oil content of macadamia nut (70–79%) [28]. It had a higher iodine value compared to macadamia nut oil and camellia seed oil. CNKO was typically characterized by a high oleic acid content of 60%, while camellia seed oil was high in α -linolenic acid and macadamia nut oil contained lauric and myristic acids [29]. Similar to olive oil, macadamia nut oil, and dragon fruit seed oil, the lipid composition in cashew nut kernel oil also consisted mainly of glycerides and phospholipids, but cashew nut kernel oil was rich in 39 phospholipid components, which was higher than the 16 reported for pitaya seed oil [16] and much less than the 172 reported for soybean, 109 for peanut, and 351 for sesame [30]. This stems from the fact these studies analyzed all phospholipid species in the fruit, whereas the present study analyzed the lipids in the oil.

Phospholipids are the main components of the cell membranes of animal and plant cells and play an important role in maintaining the physiological activity of biological membranes and the normal metabolism of the organism. They have important functions in antioxidation and delaying aging [31], regulating blood lipids and protecting the liver [32,33], and in enhancing the immunity of the organism [34,35], making them an excellent functional lipid.

Physicochemical properties and oxidative stability were important indicators of the quality of oils [22,24]. The iodine value of CNKO was (75–80) g/100 g, indicating that CNKO is a non-drying oil. The acid value was (0.41–0.53) mg/g, indicating that the content of free fatty acids in CNKO is low. The peroxide value was (0.036–0.118) g/100 g, indicating that there are less oxidation products in the oil, and the results of NIR (near-infrared) analysis indicated that the pressing temperature had no effect on the functional group structure of the oil. The above results indicate that different pressing has less effect on the quality of CNKO. In contrast, Li et al. [36] reported that there was a difference in the conclusion that the pressing process had a significant effect on the acid value and peroxide value of sesame, linseed, and perilla violet oils, and the reason for the difference might be due to the fact that the cashew nut kernels used in this study were directly pressed without roasting, which produced less antioxidant substances, such as nigrosine-like substances, due to the Merad reaction.

In addition, Michae et al. [37] showed that the oxidative stability of canola oil, olive oil, corn oil, soybean oil, sunflower oil, and flaxseed oil were 14.4 h, 19.9 h, 12.8 h, 10.9 h, 7.9 h, and 1 h, respectively, indicating that the oxidative stability of CNKO was worse than that of canola oil, olive oil, and corn oil, comparable to that of soybean oil, and better than that of sunflower oil and flaxseed oil. The oil had a high unsaturated fatty acid content and a fast oxidation rate, [38] while CNKO had an unsaturated fatty acid content up to 78%,; therefore, the oxidation of CNKO should be avoided during processing, storage, and transportation.

## 5. Conclusions

In this study, 141 lipids, including 102 glycerides and 39 phospholipids, were isolated and identified from cashew nut kernel oil using high performance liquid chromatography time-of-flight tandem mass spectrometry. Cashew nut kernel oil was a high oleic acid oil. The glycerol esters were mainly composed of DG, EtherDG, DGCC, DGGA, TG, EtherTG, and OxTG, and the phospholipids were mainly composed of LPC, LPE, LPI, PC, EtherPC, PE, EtherPE, PG, PI, and EtherPI. The total number of carbon atoms in the side chains of fatty acids in cashew nut kernel oil mass was 16–62, with a double bond number of 0–7. With the increase in pressing temperature, the functional group structure of cashew nut kernel oil was not changed, although the iodine valence, peroxide value, and the specific racemization coefficient increased, decreasing the induction time and reducing the oxidative stability of the cashew nut kernel oil.

## Figures and Tables

**Table 1:** Composition of the 102 glycerides in cashew nut kernel oil.

No.	Average Rt (min)	Average Mz	Lipid Name	Adduct Type	Formula	Ontology	Content (μg/g)
1	6.007	586.53	DG 32:0|DG 16:0_16:0	[M+NH4]+	C35H68O5	DG	43.81 ± 7.84
2	6.562	614.562	DG 34:0|DG 16:0_18:0	[M+NH4]+	C37H72O5	DG	48.39 ± 6.18
3	6.069	612.548	DG 34:1|DG 16:0_18:1	[M+NH4]+	C37H70O5	DG	900.65 ± 178.65
4	6.627	640.5787	DG 36:1|DG 18:0_18:1	[M+NH4]+	C39H74O5	DG	632.07 ± 114.33
5	6.154	638.5631	DG 36:2|DG 18:1_18:1	[M+NH4]+	C39H72O5	DG	3291.57 ± 497.25
6	5.732	636.5476	DG 36:3|DG 18:1_18:2	[M+NH4]+	C39H70O5	DG	2286.09 ± 346.24
7	5.351	634.5338	DG 36:4|DG 18:2_18:2	[M+NH4]+	C39H68O5	DG	529.40 ± 122.34
8	7.188	668.6134	DG 38:1|DG 20:0_18:1	[M+NH4]+	C41H78O5	DG	88.25 ± 12.06
9	7.175	596.5529	DG O-34:2|DG O-19:1_15:1	[M+NH4]+	C37H70O4	EtherDG	48.04 ± 4.95
10	7.241	622.5684	DG O-36:3|DG O-19:1_17:2	[M+NH4]+	C39H72O4	EtherDG	282.66 ± 26.48
11	6.786	620.5507	DG O-36:4|DG O-19:2_17:2	[M+NH4]+	C39H70O4	EtherDG	85.64 ± 7.88
12	5.612	780.6304	DGCC 36:2	[M+H]+	C46H85NO8	DGCC	935.46 ± 116.33
13	3.89	788.5853	DGGA 34:1|DGGA 16:0_18:1	[M+NH4]+	C43H78O11	DGGA	67.60 ± 9.66
14	3.924	814.6013	DGGA 36:2|DGGA 18:1_18:1	[M+NH4]+	C45H80O11	DGGA	329.67 ± 40.97
15	6.381	628.5412	TG 34:0|TG 8:0_10:0_16:0	[M+NH4]+	C37H70O6	TG	34.85 ± 1.66
16	6.943	656.5758	TG 36:0|TG 10:0_12:0_14:0	[M+NH4]+	C39H74O6	TG	40.62 ± 1.74
17	6.51	654.5604	TG 36:1|TG 8:0_10:0_18:1	[M+NH4]+	C39H72O6	TG	38.25 ± 4.56
18	7.482	684.6063	TG 38:0|TG 10:0_12:0_16:0	[M+NH4]+	C41H78O6	TG	32.71 ± 1.81
19	7.08	682.5913	TG 38:1|TG 10:0_10:0_18:1	[M+NH4]+	C41H76O6	TG	47.68 ± 5.67
20	8.019	712.6378	TG 40:0|TG 10:0_14:0_16:0	[M+NH4]+	C43H82O6	TG	27.08 ± 0.39
21	7.578	710.6234	TG 40:1|TG 10:0_12:0_18:1	[M+NH4]+	C43H80O6	TG	23.59 ± 2.48
22	8.527	740.6711	TG 42:0|TG 10:0_16:0_16:0	[M+NH4]+	C45H86O6	TG	25.50 ± 1.40
23	8.098	738.6581	TG 42:1|TG 8:0_16:0_18:1	[M+NH4]+	C45H84O6	TG	23.49 ± 1.11
24	9.016	768.703	TG 44:0|TG 14:0_14:0_16:0	[M+NH4]+	C47H90O6	TG	21.62 ± 2.27
25	8.602	766.6914	TG 44:1|TG 10:0_16:0_18:1	[M+NH4]+	C47H88O6	TG	27.25 ± 2.60
26	9.252	782.7144	TG 45:0|TG 14:0_15:0_16:0	[M+NH4]+	C48H92O6	TG	18.69 ± 2.46
27	9.503	796.7383	TG 46:0|TG 14:0_16:0_16:0	[M+NH4]+	C49H94O6	TG	30.36 ± 4.03
28	9.09	794.7215	TG 46:1|TG 12:0_16:0_18:1	[M+NH4]+	C49H92O6	TG	26.84 ± 4.20
29	8.665	792.7044	TG 46:2|TG 10:0_18:1_18:1	[M+NH4]+	C49H90O6	TG	21.53 ± 3.18
30	9.328	808.7332	TG 47:1|TG 15:0_16:0_16:1	[M+NH4]+	C50H94O6	TG	21.45 ± 3.22
31	9.919	824.7706	TG 48:0|TG 16:0_16:0_16:0	[M+NH4]+	C51H98O6	TG	173.58 ± 19.77
32	9.533	822.7522	TG 48:1|TG 14:0_16:0_18:1/TG 16:0_16:0_16:1	[M+NH4]+	C51H96O6	TG	134.10 ± 17.14
33	9.147	820.7355	TG 48:2|TG 14:0_16:0_18:2	[M+NH4]+	C51H94O6	TG	70.43 ± 13.58
34	10.135	838.7834	TG 49:0|TG 15:0_16:0_18:0/TG 16:0_16:0_17:0	[M+NH4]+	C52H100O6	TG	14.64 ± 3.10
35	9.739	836.7692	TG 49:1|TG 15:0_16:0_18:1	[M+NH4]+	C52H98O6	TG	49.24 ± 3.61
36	10.341	852.8005	TG 50:0|TG 16:0_16:0_18:0	[M+NH4]+	C53H102O6	TG	244.63 ± 22.24
37	9.934	850.7884	TG 50:1|TG 16:0_16:0_18:1	[M+NH4]+	C53H100O6	TG	10,906.80 ± 725.50
38	9.579	848.7698	TG 50:2|TG 16:0_16:0_18:2	[M+NH4]+	C53H98O6	TG	6620.33 ± 739.40
39	9.19	846.7538	TG 50:3|TG 16:0_16:1_18:2/TG 14:0_18:1_18:2	[M+NH4]+	C53H96O6	TG	691.78 ± 129.37
40	8.797	844.7354	TG 50:4|TG 14:0_18:2_18:2/TG 16:1_16:1_18:2	[M+NH4]+	C53H94O6	TG	77.47 ± 14.40
41	10.159	864.7983	TG 51:1|TG 16:0_17:0_18:1	[M+NH4]+	C54H102O6	TG	234.84 ± 34.49
42	9.785	862.7827	TG 51:2|TG 16:0_17:1_18:1	[M+NH4]+	C54H100O6	TG	316.62 ± 58.60
43	9.409	860.7709	TG 51:3|TG 15:0_18:1_18:2/TG 16:0_17:1_18:2	[M+NH4]+	C54H98O6	TG	135.56 ± 28.82
44	9.052	858.7533	TG 51:4|TG 15:1_18:1_18:2	[M+NH4]+	C54H96O6	TG	37.49 ± 8.61
45	10.728	880.8356	TG 52:0|TG 16:0_18:0_18:0	[M+NH4]+	C55H106O6	TG	245.29 ± 27.13
46	10.356	878.8196	TG 52:1|TG 16:0_18:0_18:1	[M+NH4]+	C55H104O6	TG	19,799.4 ± 1726.82
47	9.981	876.8026	TG 52:2|TG 16:0_18:1_18:1	[M+NH4]+	C55H102O6	TG	77,017.74 ± 6597.30
48	9.621	874.7887	TG 52:3|TG 16:0_18:1_18:2	[M+NH4]+	C55H100O6	TG	52,885.54 ± 4331.86
49	9.248	872.7718	TG 52:4|TG 16:0_18:2_18:2	[M+NH4]+	C55H98O6	TG	16,682.36 ± 2329.85
50	8.858	870.7551	TG 52:5|TG 16:1_18:2_18:2	[M+NH4]+	C55H96O6	TG	698.56 ± 161.73
51	8.465	868.7395	TG 52:6|TG 16:1_18:2_18:3	[M+NH4]+	C55H94O6	TG	24.58 ± 4.44
52	10.557	892.8341	TG 53:1|TG 17:0_18:0_18:1	[M+NH4]+	C56H106O6	TG	222.64 ± 42.24
53	10.198	890.8204	TG 53:2|TG 17:0_18:1_18:1	[M+NH4]+	C56H104O6	TG	978.34 ± 240.31
54	9.834	888.8024	TG 53:3|TG 17:0_18:1_18:2	[M+NH4]+	C56H102O6	TG	955.72 ± 182.39
55	9.469	886.7875	TG 53:4|TG 17:1_18:1_18:2	[M+NH4]+	C56H100O6	TG	345.10 ± 70.18
56	9.071	884.7697	TG 53:5|TG 17:1_18:2_18:2	[M+NH4]+	C56H98O6	TG	65.36 ± 15.02
57	11.079	908.8664	TG 54:0|TG 18:0_18:0_18:0	[M+NH4]+	C57H110O6	TG	166.27 ± 26.64
58	10.747	906.8533	TG 54:1|TG 18:0_18:0_18:1	[M+NH4]+	C57H108O6	TG	14,911.87 ± 2064.66
59	10.389	904.837	TG 54:2|TG 18:0_18:1_18:1	[M+NH4]+	C57H106O6	TG	71,670.86 ± 6993.73
60	10.023	902.82	TG 54:3|TG 18:1_18:1_18:1	[M+NH4]+	C57H104O6	TG	158,176.36 ± 14,506.08
61	9.664	900.8034	TG 54:4|TG 18:1_18:1_18:2	[M+NH4]+	C57H102O6	TG	113,315.23 ± 9538.59
62	9.291	898.7886	TG 54:5|TG 18:1_18:2_18:2	[M+NH4]+	C57H100O6	TG	46,748.99 ± 4593.31
63	8.915	896.7748	TG 54:6|TG 18:2_18:2_18:2	[M+NH4]+	C57H98O6	TG	9332.70 ± 2221.89
64	8.552	894.7576	TG 54:7|TG 18:2_18:2_18:3	[M+NH4]+	C57H96O6	TG	162.36 ± 28.41
65	10.588	918.8489	TG 55:2|TG 18:0_18:1_19:1	[M+NH4]+	C58H108O6	TG	120.82 ± 29.04
66	10.232	916.8322	TG 55:3|TG 18:1_18:1_19:1	[M+NH4]+	C58H106O6	TG	204.50 ± 48.06
67	11.098	934.887	TG 56:1|TG 18:0_20:0_18:1	[M+NH4]+	C59H112O6	TG	1453.44 ± 314.39
68	10.772	932.8681	TG 56:2|TG 20:0_18:1_18:1	[M+NH4]+	C59H110O6	TG	4643.38 ± 855.3
69	10.45	930.8524	TG 56:3|TG 20:0_18:1_18:2	[M+NH4]+	C59H108O6	TG	2809.19 ± 381.97
70	10.098	928.8333	TG 56:4|TG 18:1_20:1_18:2	[M+NH4]+	C59H106O6	TG	895.92 ± 118.64
71	9.725	926.8198	TG 56:5|TG 20:1_18:2_18:2	[M+NH4]+	C59H104O6	TG	169.66 ± 22.44
72	10.964	946.8829	TG 57:2|TG 21:0_18:1_18:1	[M+NH4]+	C60H112O6	TG	39.59 ± 7.56
73	11.444	962.9172	TG 58:1|TG 16:0_24:0_18:1	[M+NH4]+	C61H116O6	TG	366.37 ± 76.17
74	11.135	960.9021	TG 58:2|TG 22:0_18:1_18:1	[M+NH4]+	C61H114O6	TG	819.05 ± 196.76
75	10.826	958.882	TG 58:3|TG 22:0_18:1_18:2	[M+NH4]+	C61H112O6	TG	372.88 ± 73.15
76	10.506	956.8675	TG 58:4|TG 22:0_18:2_18:2	[M+NH4]+	C61H110O6	TG	95.71 ± 17.54
77	11.3	974.9174	TG 59:2|TG 23:0_18:1_18:1	[M+NH4]+	C62H116O6	TG	77.38 ± 17.82
78	11.002	972.8971	TG 59:3|TG 23:0_18:1_18:2	[M+NH4]+	C62H114O6	TG	45.41 ± 10.59
79	11.757	990.9497	TG 60:1|TG 18:0_24:0_18:1	[M+NH4]+	C63H120O6	TG	131.71 ± 26.08
80	11.463	988.9363	TG 60:2|TG 24:0_18:1_18:1	[M+NH4]+	C63H118O6	TG	541.34 ± 129.81
81	11.173	986.9159	TG 60:3|TG 24:0_18:1_18:2	[M+NH4]+	C63H116O6	TG	338.33 ± 80.07
82	10.875	984.9028	TG 60:4|TG 24:0_18:2_18:2	[M+NH4]+	C63H114O6	TG	90.47 ± 19.19
83	11.625	1002.948	TG 61:2|TG 25:0_18:1_18:1	[M+NH4]+	C64H120O6	TG	43.33 ± 8.71
84	11.345	1000.933	TG 61:3|TG 25:0_18:1_18:2	[M+NH4]+	C64H118O6	TG	30.67 ± 6.49
85	12.069	1018.979	TG 62:1|TG 18:0_26:0_18:1/TG 20:0_24:0_18:1	[M+NH4]+	C65H124O6	TG	16.10 ± 2.92
86	11.783	1016.962	TG 62:2|TG 26:0_18:1_18:1	[M+NH4]+	C65H122O6	TG	51.33 ± 9.60
87	11.507	1014.945	TG 62:3|TG 26:0_18:1_18:2	[M+NH4]+	C65H120O6	TG	35.72 ± 8.31
88	9.98	876.8322	TG O-53:2|TG O-17:0_18:1_18:1	[M+NH4]+	C56H106O5	EtherTG	3103.79 ± 829.83
89	9.687	874.8325	TG O-53:3|TG O-19:2_16:0_18:1	[M+NH4]+	C56H104O5	EtherTG	128.76 ± 40.46
90	9.791	888.8312	TG O-54:3|TG O-19:2_17:0_18:1	[M+NH4]+	C57H106O5	EtherTG	127.67 ± 14.35
91	9.317	898.8256	TG O-55:5|TG O-19:1_18:2_18:2/TG O-19:2_18:1_18:2	[M+NH4]+	C58H104O5	EtherTG	377.49 ± 38.47
92	8.259	864.7666	TG 50:2;1O|TG 16:0_18:1_16:1;1O	[M+NH4]+	C53H98O7	OxTG	41.54 ± 6.31
93	7.841	862.7492	TG 50:3;1O|TG 16:0_18:2_16:1;1O	[M+NH4]+	C53H96O7	OxTG	18.86 ± 1.96
94	8.734	892.7969	TG 52:2;1O|TG 16:0_18:1_18:1;1O	[M+NH4]+	C55H102O7	OxTG	101.76 ± 17.18
95	8.334	890.7819	TG 52:3;1O|TG 18:1_18:1_16:1;1O	[M+NH4]+	C55H100O7	OxTG	188.32 ± 35.24
96	7.926	888.7655	TG 52:4;1O|TG 18:1_18:2_16:1;1O	[M+NH4]+	C55H98O7	OxTG	71.32 ± 17.76
97	9.243	920.8271	TG 54:2;1O|TG 18:0_18:1_18:1;1O	[M+NH4]+	C57H106O7	OxTG	84.42 ± 12.31
98	8.784	918.8133	TG 54:3;1O|TG 18:1_18:1_18:1;1O	[M+NH4]+	C57H104O7	OxTG	206.75 ± 41.87
99	8.465	916.7983	TG 54:4;1O|TG 18:1_18:1_18:2;1O	[M+NH4]+	C57H102O7	OxTG	223.56 ± 29.44
100	8.099	914.7819	TG 54:5;1O|TG 18:1_18:2_18:2;1O	[M+NH4]+	C57H100O7	OxTG	131.72 ± 23.86
101	7.706	912.7652	TG 54:6;1O|TG 18:2_18:2_18:2;1O	[M+NH4]+	C57H98O7	OxTG	41.80 ± 6.28
102	9.702	948.8707	TG 56:2;1O|TG 18:1_18:1_20:0;1O	[M+NH4]+	C59H110O7	OxTG	25.57 ± 9.29

**Table 2:** Composition of the 39 phospholipids in cashew nut kernel oil.

No	Average Rt (min)	Average Mz	Lipid Name	Adduct Type	Formula	Ontology	Content (ng/g)
1	2.2	554.3408	LPC 16:0	[M+CH3COO]−	C24H50NO7P	LPC	71.72 ± 44.14
2	2.766	582.3713	LPC 18:0	[M+CH3COO]−	C26H54NO7P	LPC	59.52 ± 7.31
3	2.247	580.3578	LPC 18:1	[M+CH3COO]−	C26H52NO7P	LPC	452.94 ± 17.71
4	1.862	578.3447	LPC 18:2	[M+CH3COO]−	C26H50NO7P	LPC	241.56 ± 29.44
5	1.979	452.2763	LPE 16:0	[M−H]−	C21H44NO7P	LPE	55.49 ± 1.64
6	2.182	478.2912	LPE 18:1	[M−H]−	C23H46NO7P	LPE	171.82 ± 5.68
7	1.705	476.2744	LPE 18:2	[M−H]−	C23H44NO7P	LPE	51.41 ± 17.18
8	1.133	571.2898	LPI 16:0	[M−H]−	C25H49O12P	LPI	85.04 ± 8.21
9	1.242	597.294	LPI 18:1	[M−H]−	C27H51O12P	LPI	130.13 ± 10.53
10	5.58	792.5732	PC 32:0|PC 16:0_16:0	[M+CH3COO]−	C40H80NO8P	PC	174.80 ± 54.47
11	5.761	760.5845	PC 34:1|PC 16:0_18:1	[M−H]-	C42H82NO8P	PC	1947.39 ± 81.42
12	5.134	816.5735	PC 34:2|PC 16:0_18:2	[M+CH3COO]−	C42H80NO8P	PC	505.54 ± 10.96
13	6.348	846.618	PC 36:1|PC 18:0_18:1	[M+CH3COO]−	C44H86NO8P	PC	820.53 ± 70.57
14	5.619	844.6074	PC 36:2|PC 18:1_18:1	[M+CH3COO]−	C44H84NO8P	PC	3165.79 ± 125.22
15	5.173	842.5898	PC 36:3|PC 18:1_18:2	[M+CH3COO]−	C44H82NO8P	PC	1505.05 ± 52.29
16	4.781	782.5712	PC 36:4|PC 18:2_18:2	[M−H]−	C44H80NO8P	PC	201.99 ± 16.96
17	5.595	818.5919	PC O-34:2;1O|PC O-17:0_17:2;1O	[M+CH3COO]−	C42H82NO8P	EtherPC	2095.91 ± 87.63
18	6.953	846.6531	PC O-37:1|PC O-21:1_16:0	[M+CH3COO]−	C45H90NO7P	EtherPC	63.43 ± 1.90
19	4.901	690.5027	PE 32:0|PE 16:0_16:0	[M−H]−	C37H74NO8P	PE	31.97 ± 7.02
20	5.417	718.5353	PE 34:0|PE 16:0_18:0	[M−H]−	C39H78NO8P	PE	69.89 ± 19.96
21	4.955	716.5236	PE 34:1|PE 16:0_18:1	[M−H]−	C39H76NO8P	PE	1298.75 ± 15.45
22	4.621	714.5069	PE 34:2|PE 16:0_18:2	[M−H]−	C39H74NO8P	PE	248.80 ± 7.56
23	5.434	744.5578	PE 36:1|PE 18:0_18:1	[M−H]−	C41H80NO8P	PE	577.32 ± 74.51
24	5.024	742.5381	PE 36:2|PE 18:1_18:1	[M−H]−	C41H78NO8P	PE	1298.54 ± 12.70
25	4.675	740.5211	PE 36:3|PE 18:1_18:2	[M−H]−	C41H76NO8P	PE	792.68 ± 31.88
26	4.367	738.5042	PE 36:4|PE 18:2_18:2	[M−H]−	C41H74NO8P	PE	221.45 ± 2.94
27	5.01	824.541	PE 40:5;2O|PE 18:1_22:4;2O	[M−H]−	C45H80NO10P	EtherPE	74.15 ± 1.59
28	3.844	721.4987	PG 32:0|PG 16:0_16:0	[M−H]−	C38H75O10P	PG	109.15 ± 3.55
29	4.112	749.5281	PG 34:0|PG 16:0_18:0	[M−H]−	C40H79O10P	PG	67.33 ± 1.65
30	3.884	747.514	PG 34:1|PG 16:0_18:1	[M−H]−	C40H77O10P	PG	113.30 ± 3.92
31	3.672	745.4976	PG 34:2|PG 16:0_18:2	[M−H]−	C40H75O10P	PG	28.71 ± 1.90
32	3.938	773.5289	PG 36:2|PG 18:1_18:1	[M−H]−	C42H79O10P	PG	16.96 ± 4.35
33	3.805	835.5365	PI 34:1|PI 16:0_18:1	[M−H]−	C43H81O13P	PI	2347.70 ± 153.09
34	3.59	833.5206	PI 34:2|PI 16:0_18:2	[M−H]−	C43H79O13P	PI	974.35 ± 9.66
35	4.082	863.5654	PI 36:1|PI 18:0_18:1	[M−H]−	C45H85O13P	PI	587.39 ± 43.87
36	3.859	861.5489	PI 36:2|PI 18:1_18:1	[M−H]−	C45H83O13P	PI	854.96 ± 67.01
37	3.638	859.5302	PI 36:3|PI 18:1_18:2	[M−H]−	C45H81O13P	PI	401.45 ± 9.99
38	3.42	857.5183	PI 36:4|PI 18:2_18:2	[M−H]−	C45H79O13P	PI	116.70 ± 7.24
39	1.591	599.3121	PI O-18:0	[M−H]−	C27H53O12P	EtherPI	49.67 ± 5.94

**Table 3:** Effect of different pressing temperatures on the physicochemical properties of cashew nut kernel oil.

Squeezing Temperature	Acid Value (mgNaOH/g)	Iodine Value (g/100 g)	Peroxide Value (meq/kg)	Refractive Index	Specific Extinction Coefficient	
					K_232_	K_270_
100 °C	0.526 ± 0.86 ^a^	78.196 ± 17.56 ^b^	0.288 ± 0.04 ^c^	1.4612 ± 0.07 ^a^	1.027 ± 0.64 ^c^	0.114 ± 3.49 ^a^
120 °C	0.457 ± 1.46 ^b^	79.550 ± 4.34 ^a^	0.325 ± 0.11 ^b^	1.4605 ± 0.13 ^a^	1.051 ± 0.12 ^c^	0.117 ± 0.40 ^a^
140 °C	0.415 ± 0.25 ^b^	79.736 ± 29.55 ^a^	0.135 ± 0.11 ^d^	1.4623 ± 0.08 ^a^	1.007 ± 0.79 ^c^	0.053 ± 0.50 ^b^
160 °C	0.416 ± 0.00 ^b^	76.546 ± 24.19 ^c^	0.116 ± 0.13 ^d^	1.4559 ± 0.33 ^b^	1.102 ± 0.12 ^b^	0.049 ± 0.47 ^b^
180 °C	0.428 ± 0.99 ^b^	78.186 ± 41.35 ^b^	0.384 ± 0.09 ^b^	1.4578 ± 0.16 ^b^	1.154 ± 0.62 ^b^	0.080 ± 0.62 ^b^
200 °C	0.421 ± 1.55 ^b^	75.214 ± 1.44 ^d^	0.419 ± 0.11 ^a^	1.4611 ± 0.31 ^a^	1.212 ± 1.70 ^a^	0.117 ± 0.64 ^a^

Note: a, b, c, and d represented significant differences between same-column data (*p* < 0.05).

## Data Availability

Data is contained within the article.
